# Promising post-consumer PET-derived activated carbon electrode material for non-enzymatic electrochemical determination of carbofuran hydrolysate

**DOI:** 10.1038/s41598-018-31627-8

**Published:** 2018-09-03

**Authors:** Sureshkumar Ayyalusamy, Susmita Mishra, Vembu Suryanarayanan

**Affiliations:** 1 Chemical Engineering Department, National Institute of Technology Rourkela, Rourkela, 769008 Odisha India; 2 Electro Organic Division, Central Electrochemical Research Institute, Karaikudi, 630006 Tamil Nadu India

## Abstract

In this work, activated carbon (AC) materials, prepared from polyethylene terephthalate (PET) waste bottles were used as the sensing platform for the indirect detection of carbofuran. The morphology and surface properties of the PET-derived AC (PET-AC) were characterized by N_2_ adsorption/desorption isotherm, X-ray diffraction (XRD), field-emission scanning/transmission electron microscopy (FE-SEM/TEM) and Raman spectroscopy. The electrochemical activity of the PET-AC modified glassy carbon electrode (GCE) (PET-AC/GCE) was measured by cyclic voltammetry and amperometry. The enhanced surface area and desirable porosities of PET-AC are attributed for the superior electrocatalytic activity on the detection of carbofuran phenol, where, the proposed sensor shows low detection limit (0.03 µM) and remarkable sensitivity (0.11 µA µM^−1^ cm^−2^). The PET-AC/GCE holds high selectivity towards potentially interfering species. It also provides desirable stability, repeatability and reproducibility on detection of carbofuran phenol. Furthermore, the proposed sensor is utilized for the detection of carbofuran phenol in real sample applications. The above mentioned unique properties and desirable electrochemical performances suggest that the PET-derived AC is the most suitable carbonaceous materials for cost-effective and non-enzymatic electrochemical sensor.

## Introduction

Carbofuran (2,2-dimethyl-2,3-dihydro-7-benzofuranyl N-methylcarbamate) is the most extensively used carbamate pesticides in India for agriculture because of its high insecticidal activity. It is widely used for several corps such as coffee, cotton, irrigated rice, cabbage, peanut, wheat, sugarcane, maize, lettuce, potatoes, tomatoes, grapes and corn in the control of various pests^1^. As a result of excessive usage, run-off from agricultural lands, deposition from aerial application and discharge of industrial wastewater, there is a possibility for carbofuran to bioaccumulate in food and water sources. In addition, high solubility (700 mg L^−1^) and low degradability of carbofuran and its residues in aquatic environments can produce adverse effects to human beings and animals^2^. This toxin threatens human health with their inhibition activity against acetylcholinesterase (AChE), an enzyme crucial for the nerve impulse transmission in human^3^. Thus, sensitive, accurate, and rapid quantitative detection of carbofuran is essential to protect the human health and environment.

Several analytical and spectroscopic detection methods, such as, high pressure liquid chromatography (HPLC)^4^, gas chromatography^5^, mass spectrometry^6^, spectrophotometer^7^, thin-layer chromatography^8^ and fluorimetry^9^ have been developed for the determination of carbofuran in the environment. However, these techniques are often time consuming and require sophisticated apparatus, extensive labor and toxic organic reagents, making them complicated and limit its application in field routine operation. So it is essential to have fast, reliable and cost-effective technique for its detection in the environment. In recent years, the development of electrochemical methods have received considerable interest due to their benefits of minimal cost, easy operation, rapid response, compact nature, low detection limit and higher sensitivity^10^. Numerous enzymatic sensors have been reported to quantify carbofuran based on its inhibition action against the enzyme acetyl cholinesterase (AChE)^11^. However, serious drawbacks associated with enzymes such as poor stability, complicated immobilization procedures, critical operational conditions, and difficulties in handling and storing make these systems more difficult to work. Therefore, simple enzyme-free electrochemical sensor is highly desirable to alleviate the drawbacks of enzyme-based one. Several non-enzymatic sensors for carbofuran detection include cobalt oxide-reduced graphene oxide modified glassy carbon electrode^12^, hemin and nickel-graphene oxide modified carbon paste electrode^13^, screen-printed carbon electrodes modified with gold nanoparticles and graphene oxide^14^ and disposable screen-printed carbon electrode^15^. Literature studies prove that the preparations of these above materials involve complicated procedures and need complex calibration. Moreover, it cannot be utilized for on-field applications. In recent times, activated carbon (AC) has become an interesting catalytic material as electrochemical sensor due to their exclusive properties, such as increased surface area, well-developed porosity, exceptional electrical conductivity, good mechanical property and chemical stability^16–20^. Fascinatingly, the method of preparation for AC is simple, more straightforward, low cost and environment friendly when compared to the other carbon based materials.

Plastics create considerable amount of solid waste in the world on account of their application in many areas such as building, packaging, automotive, electric and electronics. Since they possess high decomposition temperature, enough resistance to ultraviolet radiation and are mostly not biodegradable, they can remain on both land and sea for several years causing environmental pollution. Hence, post utilization these plastics become waste and recovery of this ecologically hazardous waste should be taken into account instead of being left freely in nature^21^. In particular, polyethylene terephthalate (PET) bottles, being lighter, more durable and less bulky than many alternative materials find significance in the plastic industry sector. Single-use PET bottles have a short service life and therefore turn into residential (post-consumer) plastic waste in a short period of time. As a result, it would be worthwhile to find out new application areas for PET bottle wastes to maximize their end-of service life management effectively^22^. The present work deals with conversion of this waste product in to activated carbon material for the fabrication of electrochemical sensor platform

In this work, for the first time, an electrochemical sensor for indirect determination of carbofuran was fabricated based on PET derived activated carbon (PET-AC) modified glassy carbon electrode (GCE) (PET-AC/GCE). The AC had been prepared by the chemical activation of post-consumer PET bottles with potassium hydroxide (KOH). The morphology and surface properties of PET-AC were investigated by N_2_ adsorption/desorption isotherm, X-ray diffraction (XRD), field-emission scanning/transmission electron microscopy (FE-SEM/TEM) and Raman spectroscopy. Cyclic voltammetry and amperometry were used for studying the electrochemical properties of the prepared PET-AC/GCE. The experimental results suggest that PET-AC/GCE not only exhibits good selectivity, repeatability and reproducibility but also shows excellent stability for the detection of carbofuran phenol. The PET-AC/GCE provides great sensitivity and low limit of detection as an amperometric sensor, which is comparable or even superior to the results reported in the literature. The sensor also offers a noteworthy performance in the analysis of real sample.

## Results and Discussion

### Structural and textural properties of ACs

**Figure 1:**
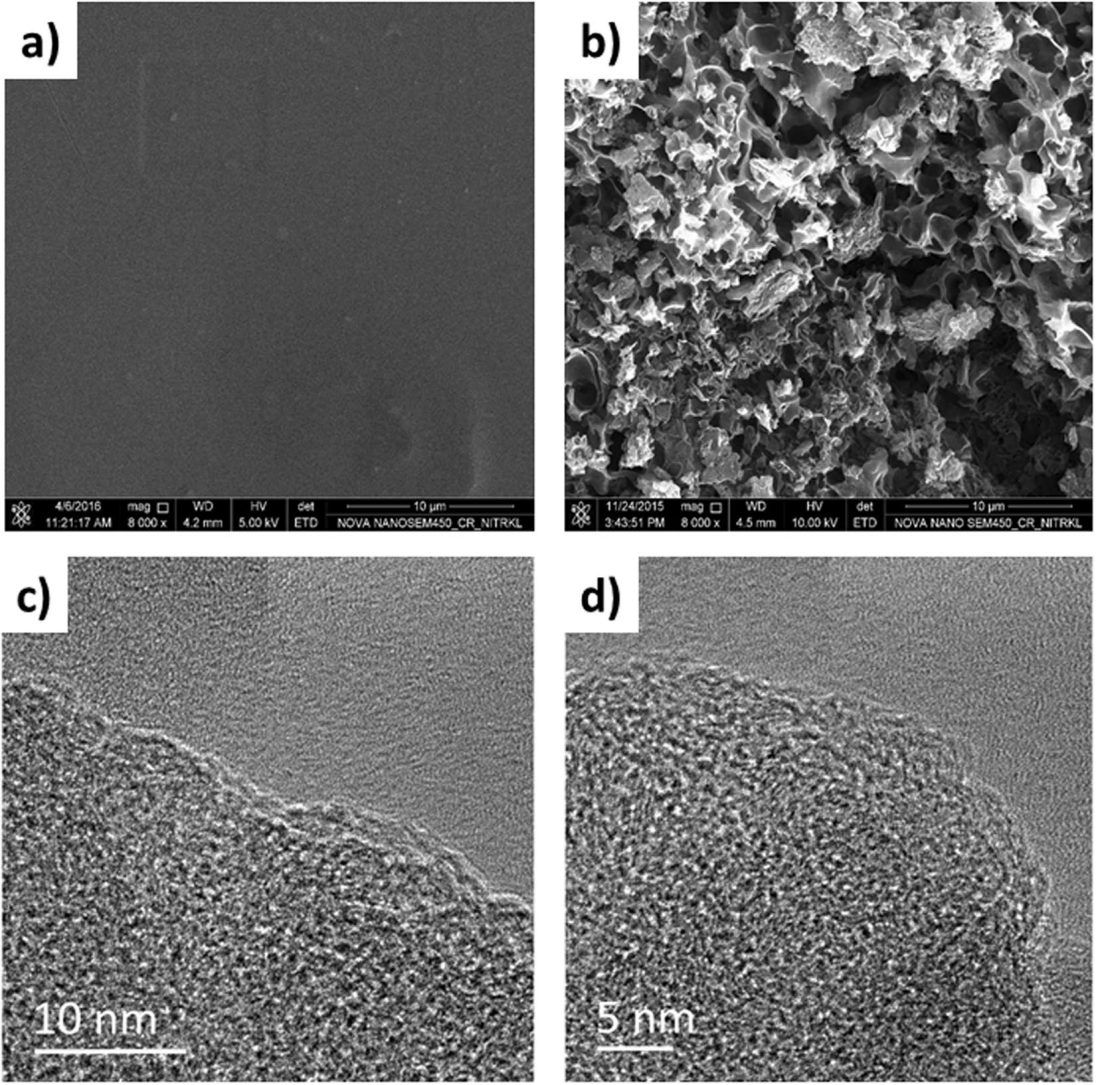
FE-SEM micrographs of (**a**) PET-RAW, (**b**) as-prepared PET-AC. (**c**) and (**d**) High resolution TEM micrographs of as-prepared PET-AC at different magnifications.

The microstructures of the raw PET and KOH treated AC are shown in Fig. 1a and b. Unlike raw-PET which shows the homogeneous and, smooth surface, KOH treated PET-AC clearly exhibits several pores with different sizes and shapes. The major surface deterioration occurs with the PET- AC owing to the discharge of volatile compounds^23^. Additional FE-TEM images of the PET-AC in Fig. 1c and d display a multi-dimensional wormhole-like pore structure^24,25^.

**Figure 2:**
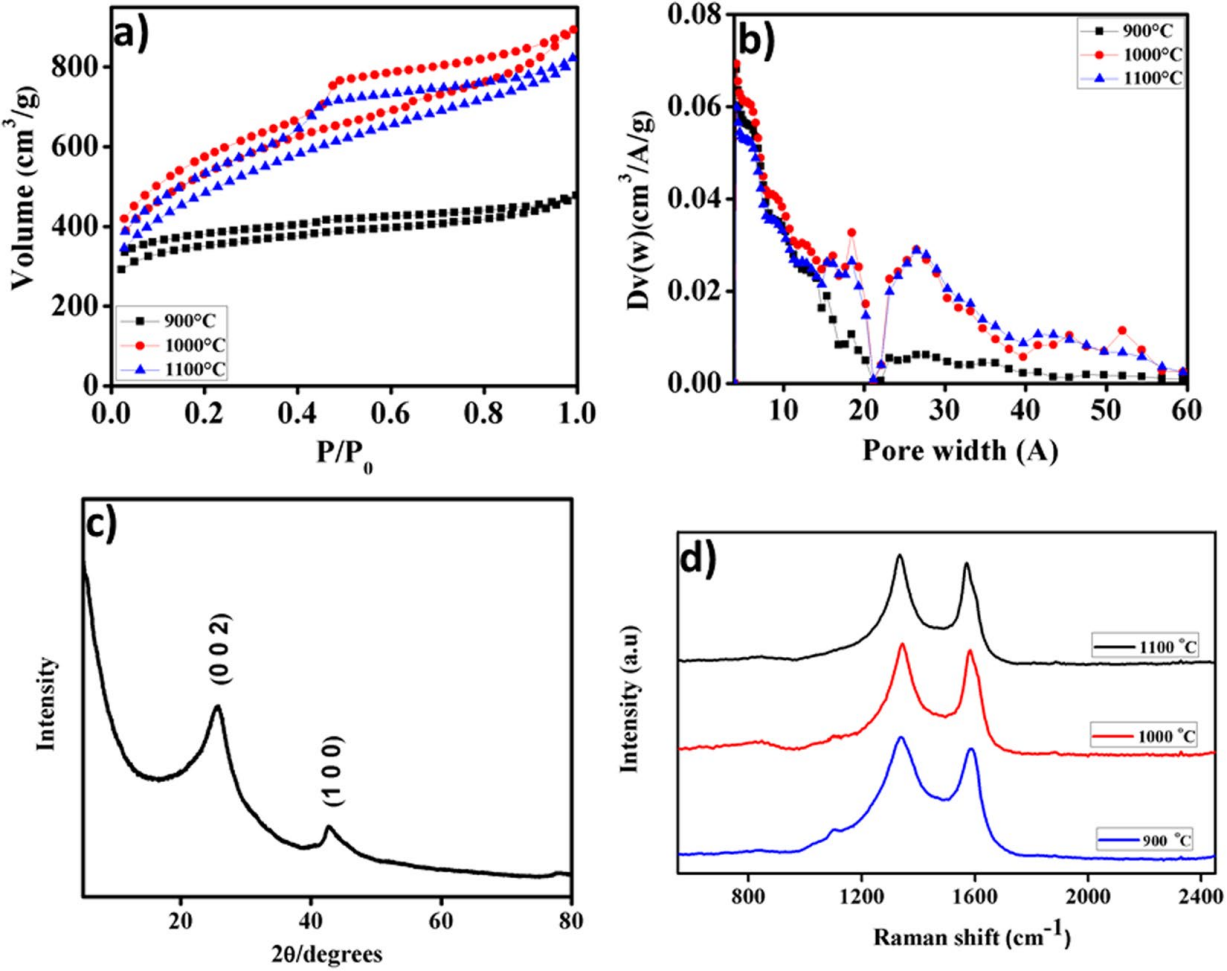
(**a**) N_2_ adsorption/desorption isotherms, (**b**) pore size distribution (**c**) XRD pattern and (**d**) Raman spectra of the as-prepared PET-AC.

Figure 2a shows N_2_ adsorption-desorption isotherms of ACs prepared at different carbonization temperatures. It is apparent that the AC prepared at low-temperature exhibits type I isotherm with substantial increase in adsorption of adsorbate below the relative pressure (*P/P*_0_ < 0.1), and a long plateau at high relative pressures, indicating the presence of microporous structure. An increase in carbonization temperature provokes an increase in the amount of N_2_ adsorbed at low relative pressure together with distinct hysteresis loop of H4 type for capillary condensation at high pressure revealing the simultaneous presence of micro and mesopores^26,27^. Thus, the isotherms belong to a mixed type in the IUPAC classification, combination of type I and type IV. Moreover, increase of the temperature from 900 to 1000 °C enhances the release of volatile matters from precursor, leading to the creation of new pores as well as widening of existing pores resulting in increment of both surface area and pore volume (Table 1). This effect is associated with the enlargement of both micropores and mesopores. However, further increase of the activation temperature to 1100 °C results in slight reduction in both surface area and pore volume. This may be due to the degradation of both pore structure and structural integrity of the activated carbon at high temperature. In general, the known chemistry of KOH activation and the development of porosity involve a chain of reactions which include dehydration, water–gas reaction, water–gas shift reaction, reduction and carbonate formation. During carbonization, KOH dehydrates to form K_2_O at around 400 °C, which further reacts with CO_2_ to form K_2_CO_3_.

1 $$2KOH\to {K}_{2}O+{H}_{2}O$$

2 $$C+{H}_{2}O\to {H}_{2}+CO$$

3 $$CO+{H}_{2}O\to {H}_{2}+C{O}_{2}$$

4 $${K}_{2}O+C{O}_{2}\to {K}_{2}C{O}_{3}$$

**Table 1:** Physical Properties of the PET-AC.

Sample	S_BET_	S_micro_	V_tot_	V_micro_	V_meso_	D_p_
PET-AC-900	1092	816	0.7387	0.4253	0.3134	2.1
PET-AC-1000	1808	883	1.382	0.4333	0.9487	3.05
PET-AC-1100	1662	667	1.272	0.3261	0.9459	3.06

S_BET_ – Brunauer-Emmet-Teller (BET) Surface area (m^2^ g^−1^).

S_micro_ – Microporous surface area (m^2^ g^−1^).

V_tot_ – Total pore volume (cm^3^ g^−1^).

V_micro_ – Micropore volume (cm^3^ g^−1^).

V_meso_ – Mesopore volume (cm^3^ g^−1^).

D_p_ – Average pore diameter (nm).

The as-formed K_2_CO_3_ is decomposed into K_2_O and CO_2_ at temperature above 700 °C. The K_2_O is reduced by carbon to produce metallic potassium at temperature over 700 °C.

5 $${K}_{2}C{O}_{3}\to {K}_{2}O+C{O}_{2}$$

6 $${K}_{2}C{O}_{3}+2C\to 2K+3CO$$

7 $${K}_{2}O+C\to 2K+CO$$

Liberated potassium metal is intercalated to the carbon matrix. Elimination of this intercalated metallic potassium by washing leads to the formation of pore structures^28,29^. The pore size distribution (PSDs) derived from DFT method is depicted in Fig. 2b. It is clear that with the exception of PET-AC-900 shows a micropore distribution; the other PET-AC samples reveal distribution of micro and mesopore sizes.

Figure 2c depicts the XRD spectrum of the as-prepared PET-AC-1000. It exhibits two broadened diffraction peaks centered at 24° and 43°, corresponding to the (0 0 2) and (1 0 0) planes, respectively. These peaks are not very intense, but well defined, indicating the negligible ordered crystalline phase. It is due to the collapse of the ordered frameworks with increase in the degree of heat treatment. These results further confirm the existence of an amorphous structure in the prepared carbon^29,30^. Figure 2d shows the Raman spectra of PET-AC taken at different temperatures. The board peak obtained at 1340 cm^−1^ corresponds to defect induced band (D) which could be concomitant to in-plane substitution as well as breaking of sp^2^ symmetry. Similarly, the first order scattering of in-plane vibration of graphitic band is observed at 1582 cm^−1^. The ID/IG ratio directly relates the index of turbostratic disorder, where in the range of 1.01 to 1.03 associates with 900 to 1100 cm^−1^ respectively. Further, as the annealing temperature increases, the G band becomes narrow due to more graphitic in nature. At the same time, the curvature of graphene like carbon nanosheets may introduce more edge oriented defects^17,29^.

### Electrochemical behavior of carbofuran phenol at PET-AC/GCE

**Figure 3:**
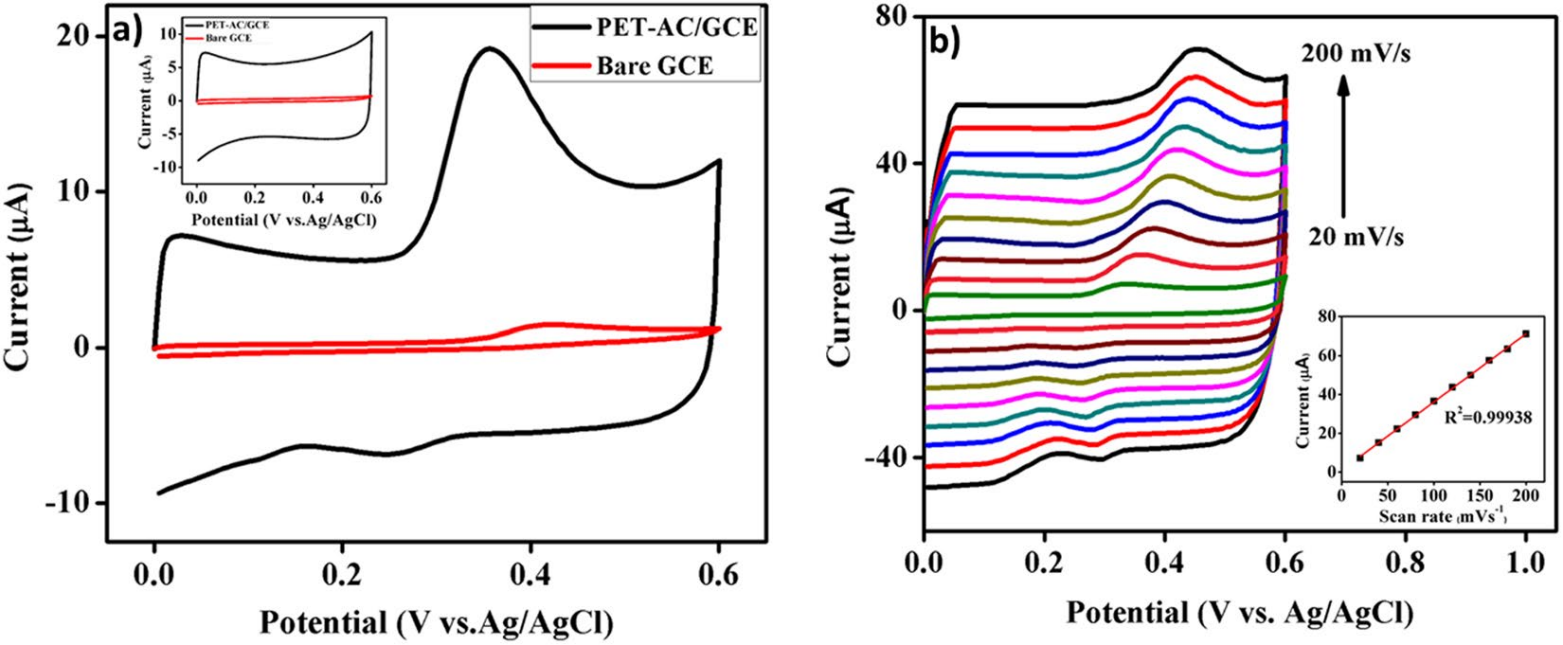
(**a**) Cyclic voltammograms recorded on bare GCE and PET-AC/GCE in the presence of 50 µM carbofuran-phenol in 0.1 M PBS (pH 7.0) at a scan rate 50 mV s^−1^. Inset: Cyclic voltammograms of bare GCE and PET-AC modified GCE without carbofuran phenol. (**b**) Cyclic voltammograms of PET-AC/GCE in 0.1 M PBS (pH 7.0) containing 50 µM carbofuran-phenol at various scan rates (20–200 mV s^−1^). Inset: plot of oxidation peak current (µA) vs. scan rate (mV s^−1^).

Cyclic voltammogram (CV) profiles of the bare GCE and PET-AC/GCE in 0.1 M PBS electrolyte (pH 7.0) with presence of carbofuran-phenol (50 µM) at a scan rate of 50 mV s^−1^ are shown in Fig. 3a and the inset shows the background voltamograms of the two electrodes. In the absence of carbofuran, PET-AC/GCE shows high background current due to the presence of non-faradaic process, when compared to bare GCE. On the other hand, PET-AC/GCE exhibits sharp and well defined anodic peak at low oxidation potential in the presence of carbofuran-phenol than that of bare GCE indicating excellent elctrocatalytic activity. Interestingly, the voltammograms recorded on the modified electrode exhibits 15 fold higher signal to background (S/B) ratio than that on GC electrode. The enhanced electrochemical activity is ascribed to the fast diffusion, good conductivity and excellent electron transfer rate of PET-AC/GCE, as a result of enriched porosity and high surface area of PET-AC^31,32^.

### Effect of scan rate

Figure 3b displays the CVs of the PET-AC/GCE at different scan rates for the electrocatalytic oxidation of carbofuran-phenol (50 µM) in 0.1 M PBS (pH 7.0). The voltammograms clearly exhibit the consistent increase in the oxidation peak current by increasing scan rate from 20 to 200 mV s^−1^, with a slight shift in oxidation peak potential toward positive direction. Furthermore, the linear variation of oxidation peak currents with scan rate (20 to 200 mV s^−1^) may be inferred by the linear regression equation: y = 0.3513 × −1.9736 with the correlation coefficient (R^2^) of 0.99938 (inset of Fig. 3b). Eventually, the results indicate that the kinetics of the oxidation peak current is controlled by the surface-controlled process^33,34^.

### Effect of pH

**Figure 4:**
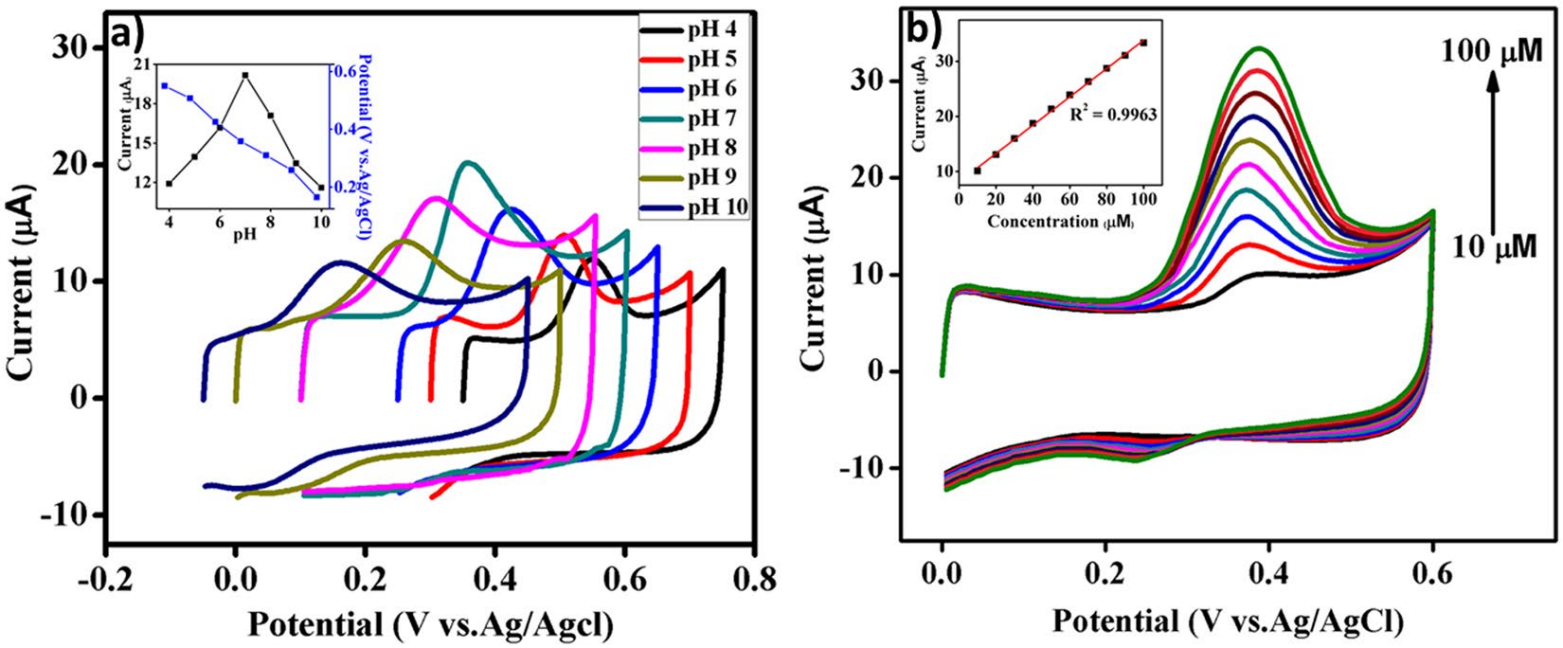
Cyclic voltammograms of PET-AC/GCE in 0.1 M PBS at a scan rate of 50 mV s^−1^ (**a**) different electrolyte pHs (from 4.0 to 10.0) with 50 µM carbofuran-phenol (50 µM). Inset: plot of peak potential (E_pa_) vs. oxidation peak current (I_pa_) vs pH. (**b**) Varied concentrations of carbofuran-phenol from 10 to 100 µM Inset: Plot of oxidation peak current (I_pa_) vs carbofuran-phenol concentration (µM).

The influence of pH on the anodic oxidation of carbofuran-phenol was investigated by varying the electrolyte pH from 4.0 to 10.0 in 0.1 M PBS at 50 mV s^−1^ scan rate in the presence of 50 µM carbofuran-phenol. As shown in the Fig. 4a, the anodic peak potential shifts negatively with the increase in electrolyte pH. Linear correlation between the anodic peak potential and buffer pH may be expressed by equation: *E*_pa_(V) = −0.0632 pH + 0.8102 (R^2^ = 0.9909). The negative slope observed in the linear equation indicates that proton transfer takes place in the electrode reaction process^35^. The inset of Fig. 4a depicts the relationship curve for pH vs I_pa_ and pH vs E_pa_. It can be seen that the oxidation peak current of carbofuran-phenol increases with increase of pH, reaches to maxima at 7.0 and then decreases. From the above investigations, a neutral pH of 7.0 is chosen optimal for the electrochemical determination of carbofuran-phenol.

### Electrocatalytic activity of PET-AC/GCE towards carbofuran-phenol determination

Figure 4b shows the electrocatalytic oxidation of carbofuran-phenol at PET-AC/GCE electrode in different concentrations of carbofuran-phenol ranging from 10 to 100 µM in 0.1 M of phosphate buffer (pH 7.0) at a constant scan rate of 50 mV s^−1^. The variation of oxidation peak currents with carbofuran-phenol concentration shows a linear relationship, which may be expressed by the linear equation (inset of Fig. 4b) I _pa_ = 0.2498x + 8.284 with correlation coefficient (R^2^) of about 0.9963 in which r is >0.99.

### Amperometric determination of carbofuran-phenol at PET-AC/GCE

**Figure 5:**
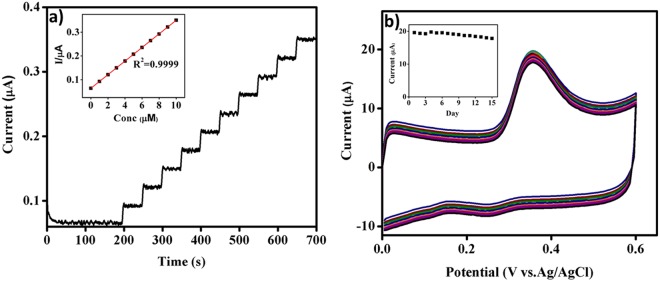
Amperometric i-t response of PET-AC/GCE at successive addition (1 µM) of carbofuran-phenol in 0.1 M PBS (pH 7.0), inset: Plot of the response current (µA) vs carbofuran-phenol concentration (µM). (**b**) Over lapped CV curves obtained for 15 days on PET-AC/GCE in 0.1 M PBS (PH 7.0) at a scan rate of 50 mV s^−1^ containing 50 µM carbofuran phenol. Inset shows the long term stability of fabricated sensor.

The amperometric responses for successive addition of 1 µM carbofuran-phenol at PET-AC/GCE in 0.1 M PBS (pH 7.0), held at a fixed potential of 0.4 V are shown in Fig. 5a. With each addition of carbofuran-phenol to the stirred supporting electrolyte solution, the current rapidly increases with the response time of less than 5 s. The current response is linear (inset of Fig. 5a) for carbofuran-phenol concentrations in the range of 1–10 µM. The linear equation is I (µA) = 0.0286x + 0.0642 with a correlation coefficient of R^2^ = 0.9999. The calculated limit of detection (LOD) is 0.03 µM and the calculated sensitivity is 0.11 µA µM^−1^ cm^−2^. Remarkably, the analytical parameters of the reported PET-AC/GCE are comparable or superior to the other reported modified electrodes available in the literature (Table 2). The stable and quick amperometric response is attributed to the high surface area and enhanced porosities of PET-AC, which clearly plays a significant role in the electrocatalytic oxidation of carbofuran-phenol.

**Table 2:** Comparison on the performance of different electrochemical methods for the determination of carbofuran by using various modified electrodes.

Electrode	Oxidation potential (V)	Linear range (µM)	Detection limit (µM)	Technique	Ref.
**AChE/Fe**_**3**_ **O**_**4**_ **-CH/GCE** ^**a**^	0.6	0.005 to 0.09	0.0036	SWV	^1^
**CoO/rGO/GCE** ^**b**^	0.4	0.5 to 70	0.02	DPV	^12^
**CPE (hemin/nickel)** ^**c**^	0.45	5 to 140	1.67	Amperometry	^13^
**Heated screen-printed carbon electrode**	—	0.4 to 400	0.05	DPV	^15^
**Au (GNPs/L-cysteine)** ^**d**^	—	0.2 to 0.67	0.18	DPV	^36^
**MIP/rGO@Au/GCE** ^**e**^	—	0.05 to 20	0.02	DPV	^37^
**PET-AC/GCE**	0.4	1 to 10	0.03	Amperometry	This work

^a^Glassy carbon electrode modified with acetylcholinesterase and iron oxide nanocomposite.

^b^Glassy carbon electrode modified with cobalt (II) oxide and reduced graphene oxide.

^c^Carbon paste electrode modified with hemin and nickel.

^d^Gold electrode modified with gold nanoparticle (GNPs) and L-cysteine.

^e^Glassy carbon electrode modified with molecularly imprinted polymer reduced graphene oxide and gold nanoparticles.

### Reproducibility, repeatability and stability

To estimate the fabrication reproducibility, six independent PET-AC/GC electrodes were tested in the presence of 50 µM carbofuran-phenol in 0.1 M PBS (pH 7.0). The measurements reveal an acceptable reproducibility with the relative standard deviation (RSD) of 3.51%. Additionally, 10 sequential measurements were carried out with RSD 3.18% to determine 50 µM carbofuran-phenol indicating the excellent repeatability of the proposed sensor. For storage stability analysis, an initially tested PET-AC/GCE was intentionally stored at room temperature and monitored for variation in the oxidation peak current over a period of about 15 days. The sensor preserves approximately 90% of its original oxidation peak current, suggesting good storage stability of the sensor (Fig. 5b and its inset).

### Selectivity of the proposed sensor

**Figure 6:**
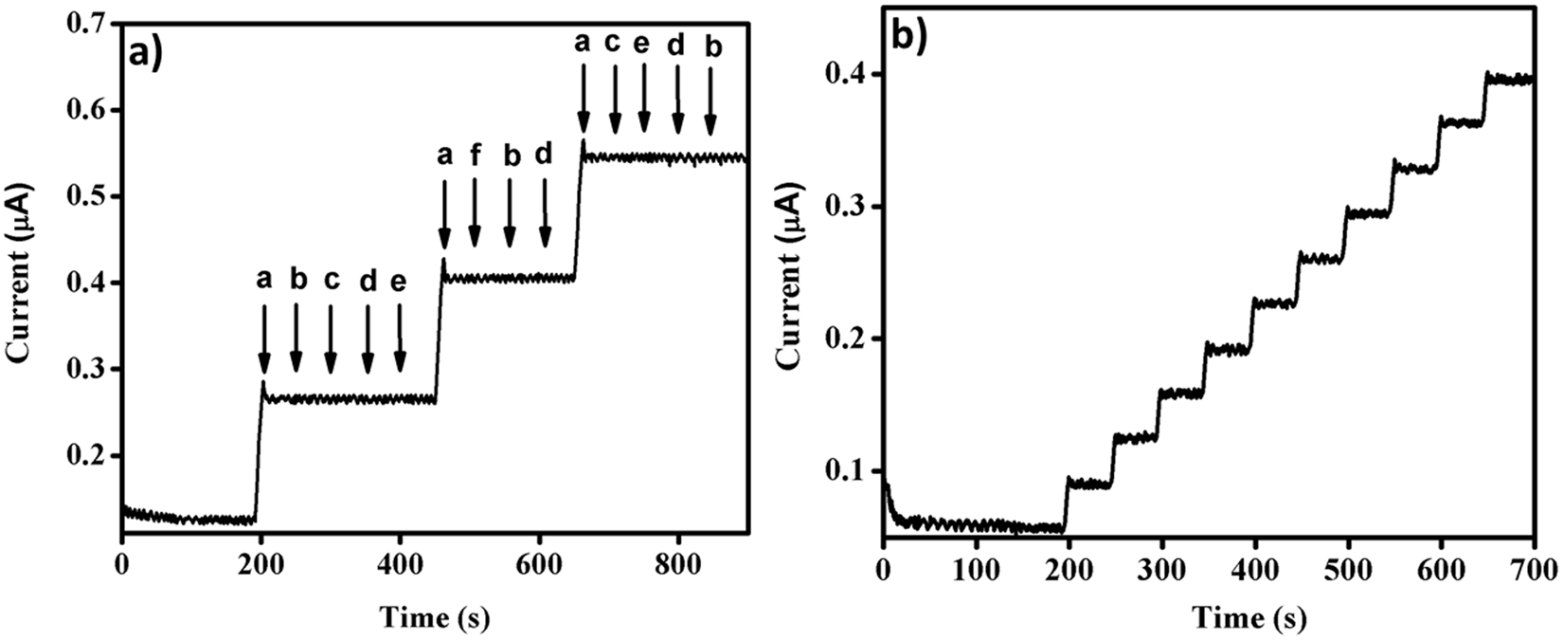
Amperometric i-t responses of PET-AC/GCE in 0.1 M PBS (PH 7.0) (**a**) selectivity test with 5 µM concentrations of (**a**) carbofuran-phenol and 50 µM concentration of (**b**) bisphenol A, (**c**) 4-nitrophenol, (**d**) glucose, (**e**) lactose, (**f**) ascorbic acid, (**b**) Real sample test with successive addition of 100 µl of real sample.

The selectivity of PET-AC/GCE electrode towards the carbofuran-phenol was studied in the presence of other potential interferences. As shown in the Fig. 6a, PET-AC/GCE electrode shows well distinct amperometric response for each addition of 5 µM carbofuran phenol (a), whereas, no noteworthy responses are observed for the addition of 10-fold excess concentration of bisphenol A (b), 4-nitrophenol (c), glucose (d), lactose (e) and ascorbic acid (f). The results clearly reveal that PET-AC/GCE possess excellent selectivity toward the detection of carbofuran phenol.

### Real sample tests

To further illustrate the practical applicability of the PET-AC/GCE, the electrode was tested with real sample, which was collected from the agricultural fields near Karaikudi, India. The amount of carbofuran in the real sample was pre-determined by UV-spectrophotometer and it was found to be 108 µM. Before the real analysis by the proposed sensor, all the carbofuran in the collected sample was converted to carbofuran phenol by hydrolyzing it in alkalescent solution at high temperature. Under the optimized conditions, 100 µl of the sample was added at regular intervals of time (50 s) in 10 ml PBS solution. For every addition of collected sample, a quick response of 0.034 µA current is observed (Fig. 6b). From the amperometry results, the concentration of collected real sample is found to be 120 µM. The results show that the PET-AC/GCE could be efficiently applied for real sample analysis with good accuracy.

## Conclusions

In summary, activated carbon with high surface area and good porosities were successfully prepared from post-consumer PET bottles by simple and cost-effective chemical activation method with KOH. The materials were characterized by N_2_ adsorption/desorption isotherm, XRD, FESEM, TEM and Raman spectroscopy. The fabricated PET-AC/GCE exhibits enhanced electro catalytic activity as non-enzymatic electrochemical sensor for the trace level determination of carbofuran phenol, as assessed by CV and amperometry. Notably, the PET-AC/GCE shows excellent detection limit of 0.03 µM and ultrahigh sensitivity 0.11 µA µM^−1^ cm^−2^ for the detection of carbofuran phenol. The proposed sensor is a good alternative to the conventional GCE and most other previously reported modified electrodes, as it offers superior stability, sensitivity, detection limit, reproducibility and selectivity. Furthermore, the amperometric sensor provides remarkable results in real sample applications.

## Experimental Section

### Materials and Chemicals

Post-consumer PET bottles were collected from the premises of National Institute of Technology Rourkela, India. Analytical grade carbofuran and potassium hydroxide (KOH) were commercially obtained from Sigma-Aldrich and Merck respectively. The supporting electrolytes (phosphate buffer solution (PBS)) at pH 7.0 were prepared using 0.05 M Na_2_HPO_4_ and NaH_2_PO_4_ solutions and the pH was adjusted with 0.1 M H_3_PO_4_ and 0.1 M NaOH. All other chemicals used were of analytical reagent grade and were used as received without further purification. All experiments were performed with ultrapure water (Millipore) at room temperature.

**Figure 7:**
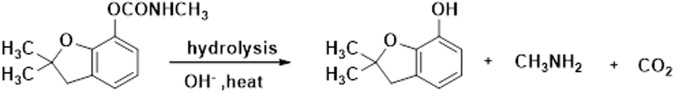
Hydrolysis of carbofuran to carbofuran phenol.

According to earlier research, carbofuran with limited electrochemical activity could be converted into carbofuran-phenol by hydrolyzing it in alkaline solution to increase electrochemical activity, where its complete conversion was ensured^12,15^. Carbofuran solution of 1.0 × 10^−3^ M was prepared by dissolving 22 mg of the compound in 100 ml of 0.1 M NaOH solution and heated for 1 h to hydrolyze all the carbofuran to carbofuran phenol (Fig. 7)^15^.

### Preparation of PET-AC material

Raw material, PET, was properly cleaned to remove the impurities and then dried in oven to remove the moisture content. The dried sample was chopped to a particle size range of 5–10 mm. Then, 10 g of PET granules were put in 100 ml of dilute KOH with an impregnation ratio 5. It was kept at 85 °C for 24 h, followed by drying at 120 °C for 4 h. The resultant samples were carbonized in a horizontal tubular furnace at final temperature in the range from 900 °C to 1100 °C with a definite heating rate (10 °C/min) in N_2_-atmosphere (100 ml/min). The samples were kept for 60 min at the final temperature. After cooling, the products were rinsed with 0.1 M HCl and hot water (80 °C) until the pH became neutral. Finally, The rinsed carbons were dried at 120 °C for 24 h in an oven and stored in air tight container.

### Preparation of AC-modified electrode

As prepared PET-AC (5.0 mg) was dispersed in 1 ml ethanol, under sonication for 3 h. Meanwhile, the surface of the GCE was mirror polished with 0.3 and 0.05 µm alumina powder and ultra-sonicated for several minutes with ethanol and ultrapure water before modification. An aliquot of 5 µl ethanol/PET-AC suspension was introduced onto the surface of GCE using the drop casting method, followed by drying at 50 °C for 2 h. Subsequently, the PET-AC/GCE was gently rinsed with ultrapure water again to remove loosely bound ACs. Finally, the fabricated PET-AC/GCE was explored as the working electrode for further electrochemical measurements.

### Characterization techniques

X-ray powder diffraction (XRD) experiment was carried out in Rigaku ultima IV diffractometer equipped with Cu Kα radiation. The surface morphologies of the ACs were studied using field emission scanning electron microscope (FE-SEM) (FEI, Nova NanoSEM 450) & transmission electron microscope (TEM) (FEI, Tecnai S-TWIN). Raman spectrum was obtained with BRUKER RFS 27 stand-alone laser Raman spectrometer using 1064 nm Nd:YAG laser source at a spectral range of 300–2500 cm^−1^. The N_2_ adsorption-desorption isotherms of the AC were analyzed at 77 K using Quantachrome (Autosorb-1) surface area analyzer. Prior to effecting adsorption measurements, samples were outgassed overnight at 200 °C under helium. The apparent surface area was derived according to the BET (Brunauer-Emmet-Teller) method. The pore size distribution of AC was calculated by Density functional theory (DFT) method. All electrochemical experiments were conducted using electrochemical work station (Autolab PGSTAT 30, Eco Chemie, Netherlands). A conventional three electrode setup was utilized using bare and modified GCE as the working electrode, Ag/AgCl (in saturated KCl) as the reference electrode and a large platinum (Pt) foil as the counter electrode. All electrochemical experiments were carried out under inert atmosphere at room temperature.
